# The Impact of Aging Drivers and Vehicles on the Injury Severity of Crash Victims

**DOI:** 10.3390/ijerph192417097

**Published:** 2022-12-19

**Authors:** Miguel Santolino, Luis Céspedes, Mercedes Ayuso

**Affiliations:** 1 Department of Econometrics-Riskcenter-IREA, University of Barcelona, 08034 Barcelona, Spain; 2 Zurich Insurance and Riskcenter-IREA, 08034 Barcelona, Spain

**Keywords:** traffic crashes, severity, dependence, random effects, driver age, vehicle age

## Abstract

Against a general trend of increasing driver longevity, the injuries suffered by vehicle occupants in Spanish road traffic crashes are analyzed by the level of severity of their bodily injuries (BI). Generalized linear mixed models are applied to model the proportion of non-serious, serious, and fatal victims. The dependence between vehicles involved in the same crash is captured by including random effects. The effect of driver age and vehicle age and their interaction on the proportion of injured victims is analyzed. We find a nonlinear relationship between driver age and BI severity, with young and older drivers constituting the riskiest groups. In contrast, the expected severity of the crash increases linearly up to a vehicle age of 18 and remains constant thereafter at the highest level of BI severity. No interaction between the two variables is found. These results are especially relevant for countries such as Spain with increasing driver longevity and an aging car fleet.

## 1. Introduction

Road traffic crashes constitute a major public health concern worldwide. Approximately 1.3 million people die each year as a result of traffic accidents, and between 20 and 30 million people suffer non-fatal injuries [1]. To design effective strategies to limit the number of victims of such events, we need a better understanding of the risk factors affecting the likelihood of being involved in a road crash and of its severity. This study seeks to take a step in that direction.

Our aim here, therefore, is to analyze the road crash risk factors that affect the expected proportion of bodily injury (BI) victims by level of severity. To do so, this paper analyzes the injuries suffered by vehicle occupants in traffic crashes on Spain’s roads using a BI severity level. We use official police data recording crashes involving victims in 2016 and examine a series of risk factors associated with the vehicle, the driver, and the crash itself considered as having a significant impact on the expected proportion of vehicle occupants suffering non-serious (slight), serious, and fatal injuries in a crash. Identifying the factors that affect road safety and understanding the impact of different vehicle attributes should help in the development of new safety features and improved transportation safety programs. In our analysis of factors that affect the expected number of injured occupants by level of BI severity, we pay particular attention to driver age, vehicle age, and the interaction of the two.

The contribution of this study is threefold: First, the methodology we employ is able to capture the heterogeneity attributable to the involvement of more than one vehicle in the same crash; second, any prior form of association between driver age and vehicle age, on the one hand, and the expected severity of the motor crash, on the other, is not stated, but rather is determined by the data; and, third, the potential interaction between driver age and vehicle age that might enhance the impact on expected BI severity is fully investigated.

A number of studies indicate that the effect of driver age is nonlinear both with crash severity—with young and old drivers constituting the riskiest groups [2,3,4]—and with the probability of causing a crash [5]. Recent research efforts have focused on older drivers, given their increasing longevity and the impact of aging on road traffic crash injury rates [6,7,8]. Researchers have also been interested in the shape of the relationship between crash severity and vehicle age. Here, the accelerated incorporation in recent decades of technological safety improvements in newer vehicles means the latest generations of cars are associated with lower probabilities of injury and fatality in road crashes [9,10,11,12,13,14]. Narváez-Villa et al. [15] report that drivers of all ages reduce their mileage as their vehicles age, suggesting that at equal exposure, the probabilities of crashes and, therefore, of potential injury are even smaller. Against a backdrop of increasing longevity, and with drivers driving until later ages, a certain association might also be expected between the aging of the driver and the aging of their vehicle, reflecting a lower expected tendency to change vehicles after a certain age. As such, an association between these two factors and the severity of injuries suffered in a traffic accident can be expected. Whereas Ayuso et al. [12] reported an increase in the probability of fatal and serious injuries in drivers over the age of 75 and in vehicles older than the average age of the car fleet, here we aim to capture the existence of this interaction in more global terms, first, by demonstrating whether longevity in drivers and aging vehicles are statistically correlated, and, second, by measuring whether the simultaneous inclusion of the two variables as a covariate has a significant effect on the probability that the injuries resulting from a crash present a certain level of BI severity.

A vast number of studies, conducted from a range of different perspectives, have analyzed the risk factors that affect BI severity following a road traffic crash. Some focus on the type of vehicle involved and the resulting injuries [16,17,18,19]. For instance, two-wheeled motor vehicles are associated with a greater risk of serious injury or fatality [20,21,22,23], whereas heavier vehicles cause more damage to other vehicles but provide better protection for their occupants [24]. Other studies have examined differences between crash type and BI severity, the latter increasing, for example, when the accident involves a frontal rather than rear impact [25]. In rollover crashes and drops, passengers are more likely to suffer serious head and cervical spine injuries [26,27]. Similarly, analyses show that driving under non-optimal conditions of light and on non-optimal road surfaces play an important role in crash severity [28,29,30]. However, as Eluru et al. [31] indicate, there is strong evidence of the presence of correlated unobserved factors affecting BI severity levels among vehicle occupants.

Some authors have used random parameter models to account for the heterogeneity attributable to unobserved factors related to road geometrics, vehicle types, and spatial areas [32,33,34]. Anastasopoulos and Mannering [35] suggest that ignoring the possibility of random parameters when estimating count data models can result in changes to the magnitude of the effect of factors impacting crash frequency. Anastasopoulos and Mannering [36] draw a similar conclusion when demonstrating that random parameter models using less detailed crash-specific data are still able to provide a reasonable level of accuracy. Osman et al. [37] argue that injury severity conditional on crash occurrence can depend on numerous factors, none of which are included in crash databases. They go on to stress that the unobserved heterogeneity derived from these unobserved factors can moderate the influence of other observed covariates in the model, leading to variation in the parameter effects across different observations. Finally, Hosseinpour et al. [38] estimate crash counts for four multi-vehicle collision types and report dependencies between collision types and a spatial correlation between adjacent sites.

In this paper, we seek to further analyze the dependencies between driver and vehicle ages and BI severity in road traffic accidents. In so doing, we also include the effect of unobserved factors that might influence the correlation between the two variables. Previous studies show that older drivers drive older vehicles more frequently [6,12]; here, we aim to test whether this correlation is statistically significant in explaining differences in crash severity. We apply generalized linear models (GLMs) with random effects—that is, generalized linear mixed models (GLMMs)—to examine the dependence between vehicles involved in the same crash. Specifically, we apply a binomial regression model with random effects, which is a particular case of random parameter models. By including random effects in fixed linear models, we are able to analyze multilevel data when those data have more than one source of random variability. As Mannering et al. [32] point out, multivariate issues are likely to arise in the case of crashes involving multiple occupant injuries incurred in the same accident. In such instances, unobserved factors that influence the severity of the injuries—such as the structural characteristics of the vehicles involved, among others—would be correlated [31,32,33]. Indeed, the structural characteristics of new- and older-generation vehicles can vary considerably. The GLMM framework assumes a linear relationship between the dependent variable and the covariables. Additionally, a semiparametric GLMM is fitted to the data to determine the real form of dependence between driver and vehicle ages and the severity of the motor traffic crash.

The rest of this paper is structured as follows. Section 2 defines the GLMM used to model the proportion of injured victims in a crash by level of BI severity when including random effects. Section 3 describes the dataset and presents the key descriptive statistics. Results related to the model selection and the binomial GLMM estimated are reported in Section 4, where a detailed analysis of the impact of driver age and vehicle age on BI severity is carried out. Discussion is provided in Section 5, and Section 6 concludes.

## 2. Generalized Linear Mixed Models

Our analysis focuses on the relationship between a set of risk factors and the number of victims in a vehicle involved in a crash according to the severity of their injuries. We deal with three discrete variables: the number of non-seriously injured occupants in the vehicle, *y*^ns^; the number of seriously injured occupants, *y*^s^; and the number of fatally injured occupants, *y*^f^, where injuries are considered non-serious if the victim suffered only minor personal injuries and did not require hospitalization or was hospitalized for less than 24 h; serious if they required hospitalization for more than 24 h; and fatal if the victim’s death occurs as a result of the crash within a 30-day period following the accident. The unit of observation in our analysis is the vehicle involved in the crash.

The number of injured victims is a function of vehicle occupancy. The set of vehicles included in the analysis has different passenger capacities and, even if they had the same capacity, the number of occupants at the time of the crash is likely to differ. The number of injured occupants per level of BI severity are modeled in relative terms, i.e., the proportion of injured victims in relation to the total vehicle occupancy. GLMs with a binomial error distribution is the appropriate regression when the dependent variable is expressed in relative terms. The GLM relates the conditional mean of the distribution µ and the linear regression through the link function g as follows: $g\left({µ}_{i}\right)=\eta_{i}=x_{i}^{ᵀ}\beta$ for the *i*th vehicle, *i* = 1,…, *I*, where $\eta_{i}$ is the linear predictor, *β* is the vector of the regression coefficients, and $x_{i}$ is the vector of regressors. The dependent variable $y^{j}$ reflecting the relative number of injured victims in the vehicle according to the level of severity *j* = (ns,s,f) follows a binomial distribution, $y^{j}\;\sim B\left(s,\pi_{j}\right)$, where *s* is the number of occupants in the vehicle and $\pi_{j}$ is the proportion of victims injured with a severity level *j*. If the canonic link function selected is $ln\left(\frac{\pi_{j}}{1-\pi_{j}}\right)$, the binomial specification is equivalent to the logit regression model [38].

When multiple vehicles are involved in a crash, the number of victims presenting the same level of BI in each vehicle is assumed to be correlated [31]. When a dataset presents correlated clusters, GLMMs are a more appropriate specification. GLMMs are an extension of GLMs that incorporate random effects for the analysis of multilevel data. Now, we introduce a Q-dimension vector of cluster-specific parameters $\theta_{n}=\left(\theta_{n1}\;,.\;.\;.\;,\;\theta_{nQ}\;\right)$ and a vector $z_{ni}$ of predictors corresponding to the random effects, for n = 1,…,N. In our case, *n* indicates the crash and only one cluster-specific parameter is considered, so $\theta_{n}$ and $z_{ni}$ are scalars. In the GLMM with a cluster-specific variable, the conditional mean $\mu_{ni}$ is regressed on the predictors as follows: $g\left({µ}_{ni}\right)=x_{ni}^{ᵀ}\beta +z_{ni}^{}\theta_{n}$. The constant term of the linear predictor is no longer the same for all observations but now varies for each group of vehicles involved in the same crash. Thus, unobserved individual-specific heterogeneity associated with the crash in which the vehicle was involved is introduced into the regression modeling.

## 3. Data

The dataset of road crashes involving victims was provided by the Spanish Traffic Authority (DGT). It contains information monitoring the evolution of victims in a thirty-day period following the accident, as recorded by traffic agents. The complete database contains information for 100,494 police-reported motor vehicle crashes with victims for the period from January 2016 to December 2016. A total of 179,295 vehicles were involved, there being no victims in 73,611 of the vehicles and at least one victim in 105,684 of the vehicles. Only those vehicles presenting complete records in line with our research requirements were selected. Thus, we analyzed 96,472 vehicles involved in 59,040 crashes (Table 1). Of these, 46.67% involved one vehicle, 45.88% involved two, and the remaining 7.45% involved more than two vehicles. In 42.27% of the vehicles, none of the occupants were injured as a result of the crash, whereas in 57.73%, at least one occupant was injured.

Table 2 presents the variables used in our analysis. The dataset contains information on the number of victims in each vehicle by level of BI severity level, differentiating between i) non-injury, ii) non-serious or slight injury, iii) serious injury, and iv) fatalities. Driver information includes age and gender. Vehicle information includes type, age, and number of occupants (including the driver). Other variables related to the accident, that is, crash type, road type, road conditions, and visibility, are also included.

The mean age of the drivers involved in the crashes was 41.4, and the mean vehicle age was 10.35. The mean number of occupants per vehicle was 1.41. Most occupants suffered non-serious injuries (average of 0.69 per vehicle), followed by occupants who did not suffer any injuries (average of 0.65 per vehicle), serious injuries (average of 0.06 per vehicle), and fatalities (average of 0.01 per vehicle).

**Figure 1:**
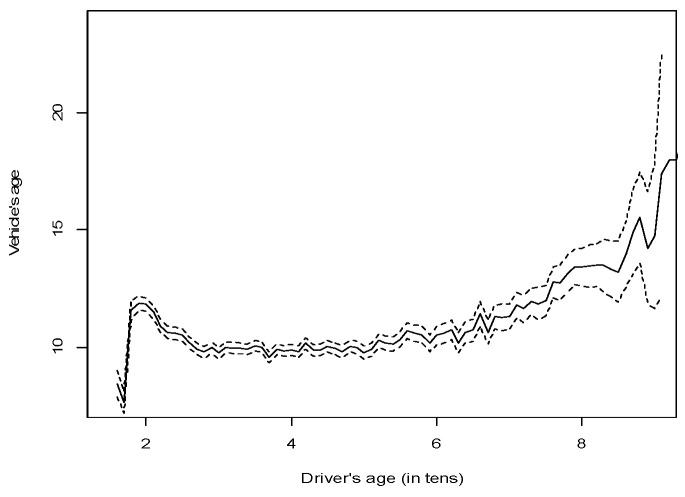
Mean (95% CI) vehicle age by driver age in Spanish road traffic crashes.

The association between driver age and vehicle age was evaluated. Pearson’s correlation and rank-based measures of association between the two variables were computed and no significant association was found (Pearson’s correlation: 0.043; Spearman Kendall’s τ: 0.008, and Spearman’s ρ: 0.012). We next tested for an association between the mean age of the vehicle and the age of the drivers, respectively (Figure 1). Although no association was detected, Figure 1 seems to indicate that the mean vehicle age increased in the case of drivers over the age of 65. Association measures were again computed conditioned specifically to drivers over 65 but the values increased only slightly (Pearson’s correlation: 0.152; Spearman Kendall’s τ: 0.101, and Spearman’s ρ: 0.143). Thus, we conclude that no relevant association between the age of the driver and the age of the vehicle was detected. As is evident from the confidence intervals shown in Figure 1 (dashed lines), dispersion around the mean age of the vehicle increased with the age of the driver, which could affect the results obtained.

Figure 1 also shows that younger drivers (probably reflecting the fact that drivers in the first few years after obtaining their license drive non-new vehicles) and older drivers tend to drive older vehicles.

## 4. Modeling the Proportion of Occupants Injured by BI Severity

### 4.1. Model Selection

Three binomial regression models were compared to model the proportions of non-seriously, seriously, and fatally injured occupants. GLMs were, first, fitted to the binomial distribution. Second, to capture the dependence between the vehicles involved in the same crash, a GLMM with a random effect was fitted to the data. The Akaike information criteria (AIC) and the Bayesian information criteria (BIC) for the three GLMs and GLMMs are presented in Table 3. Coefficient estimates of GLMs and GLMMs are reported in Appendix A. The random effects binomial presents the lowest AIC and BIC for each of the models considered, which suggests that when the random effect is included, the model is a better fit and captures the correlation between vehicles in the same car crash.

### 4.2. Relationship between Vehicle Age, Driver Age, and BI Severity

**Figure 2:**
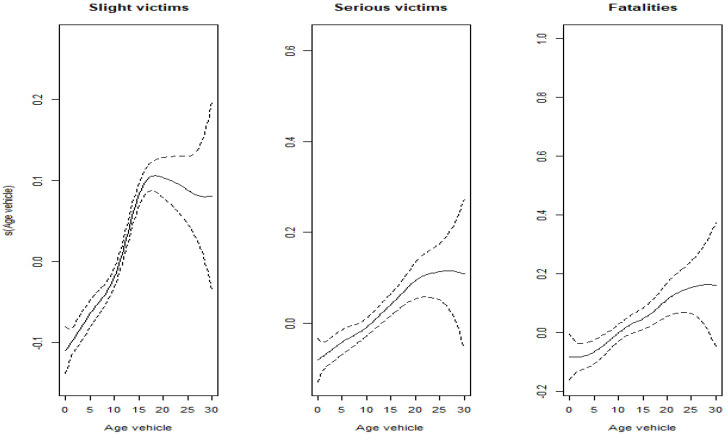
Estimated effect of vehicle age in the semiparametric binomial regression, by BI severity.

Both the GLMM and GLM frameworks assume that the relationship between the continuous variables and the transformed dependent variable is linear. However, this will not always be the case. Here, we investigate the relationships between vehicle age and driver age, respectively, and the severity of injury sustained by occupants of the vehicle involved in the crash. To do so, a semiparametric binomial regression is fitted to the data. These flexible modeling approaches define the linear predictor as a linear relationship between the categorical variables and smooth functions of the two continuous variables, that is, *Vehicle age* and *Driver age*. Although this estimation process is frequently less stable and the coefficient interpretation more complex, flexible modeling is a powerful tool for understanding the effect of the explanatory continuous variables on the dependent variable in a multivariate context. Figure 2 shows the estimated effect of vehicle age on the linear predictor of the binomial regression model. Coefficient estimates of the semiparametric binomial regression model are reported in Appendix B. There is an appreciable shift in this trend at a vehicle age of around 20 in the case of victims suffering a non-serious injury; however, the effect is less apparent for victims suffering serious and fatal injuries, where we find an increasing behavior that tends to stabilize at maximum values after the age of 20.

**Figure 3:**
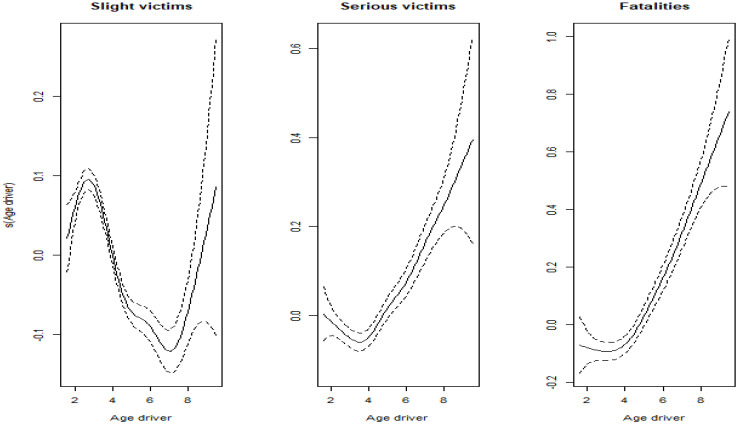
Estimated effect of driver age in the semiparametric binomial GLMM, by BI severity.

We conducted the same analysis for driver age (see Figure 3) and obtained a quadratic shape for victims with serious and fatal injuries. A more complex relationship is observed in the case of victims suffering only a slight injury, where a quadratic shape does not capture the initial increase up to the age of around thirty.

Different transformations of the two explanatory variables, including polynomials and linear approximations, were analyzed to capture the relationships shown in Figure 2 and Figure 3. Eventually we opted for the transformation associated with the lowest AIC when the model is fitted. It is not shown here for reasons of simplicity. In the case of vehicle age, the variable is replaced by two new regressors: *Young vehicle*, a quantitative variable with a continuous part for those vehicles under the age of 18, taking the value of the vehicle age when it is under 18 and 0 otherwise; and, *Old vehicle*, defined as a dichotomous variable taking the value 1 if the vehicle is 18 or older and 0 otherwise. In the case of driver age, a quadratic shape provided the best fit for the three BI severity levels, including the number of non-serious injury victims. Thus, a new regressor is added to the model to record the squared age of the driver (*Squared age*).

### 4.3. Binomial Generalized Linear Mixed Model

A binomial GLMM was fitted for the proportions of victims presenting slight, serious, and fatal injuries in the vehicle, including the new regressors of vehicle age and driver age. Table 4 shows the estimated coefficients for the three binomial GLMMs. A negative (positive) coefficient indicates a decrease (increase) in the expected proportion of victims with non-serious, serious, or fatal injuries in the vehicle, respectively.

Driver age has an impact on the expected proportion of all victims. The expected proportion of victims decreases with increasing driver age until a minimum is reached, and then increases. This holds for all victim types. In the case of victims with slight injuries, the minimum is reached at the age of 91; for those with serious injuries, at the age of 31; and in the case of fatalities, at the age of 24. Driving old vehicles increases the expected proportion of injured occupants. This proportion increases each year of additional vehicle age up to 18, when the effect remains stable at the highest level for vehicles of 18 years and more, mainly for seriously injured victims and fatalities. However, the covariate reflecting the interaction between driver age and vehicle age does not present a significant coefficient in any of the regression models, which prevents us from speaking of a clear joint or simultaneous effect of the two variables.

When the driver involved in the crash is male, the expected proportion of seriously injured occupants and fatalities increases while the proportion of slightly injured occupants falls. If we take cars as our reference, the expected number of injured occupants increases for all three BI severities when a crash involves a two-wheeled vehicle, that is, motorcycles, mopeds, and bicycles. In the case of vans and heavy vehicles, the number of slightly injured victims is lower than in cars, but no significant differences are found in relation to the number of seriously and fatally injured victims. Illumination, road surface conditions, and road type are significant factors in explaining the number of injured occupants. Driving in non-optimal conditions of visibility increases the expected number of all types of injured victims. When road surface conditions are non-optimal, however, although the number of slightly injured victims increases, the numbers of seriously injured victims and fatalities fall.

Principal and minor roads are associated with a higher expected number of injured occupants than local roads. The estimated coefficients for minor roads are slightly higher than those for principal roads, regardless of BI severity. Thus, the expected number of injured victims is higher on minor than on principal roads. Finally, when the crash involves a pile-up or a run-over, the expected number of injured victims in the vehicle falls compared to collisions involving other vehicles, collisions with an obstacle, rollover crashes, or drops.

## 5. Discussion

This study analyzed several road crash risk factors that affect the expected proportion of BI victims by level of severity, taking into account the dependence between the vehicles involved in the same crash. The observation unit employed in this analysis is the vehicle, and we consider the dependence between vehicles involved in the same crash, including its random effects in the regression. The model performance is found to improve when this dependence is taken into consideration. Thus, the inclusion of random effects captures, at least partially, the heterogeneity due to the involvement of more than one vehicle in the same crash.

When two or more vehicles are involved in the same crash, we might expect to derive a relationship between the damage they suffer respectively and the severity of injury of the victims. Several studies report the incidence and severity of injuries when different types of vehicle are involved, including passenger vehicles and trucks [39,40] and motorbikes and non-motorbike vehicles [41], as well as the position of occupants inside the vehicle [42,43]. Dependence between the BI severity levels of those involved in the same crash can be especially relevant if we seek to predict the expected number of victims and their injury severity; for example, as a consequence of a safety policy or, more specifically, in the insurance context, when we wish to calculate provisions for the coverage of automobile claims. Methodologically, this objective is in line with previous studies that suggest that ignoring the possibility of including random parameters when estimating count-data models may affect the magnitude of the coefficients [35,36,37,38].

Our analysis pays special attention to the age of the driver and vehicle age as factors explaining the proportion of occupants presenting different levels of BI severity in a crash. We demonstrate that the relationship between these factors and the (transformed) dependent variable is nonlinear. Subsequently, both factors were redefined to reflect their association with the expected proportion of injured occupants.

In the case of the age of the driver, we found a quadratic relationship with the severity of injury of vehicle occupants. Indeed, in line with previous studies [4,44,45], young and old drivers constituted the riskiest groups. Young drivers were associated with a high risk of accidents with non-serious injuries, whereas old drivers presented the highest risk in accidents with serious and fatal injuries. This does not, however, mean that older drivers are necessarily more dangerous drivers; rather, it seems to reflect the fact that older drivers (and their old passengers, too) are inherently more likely to be seriously injured in crashes due to physical frailty [4,46]. Previous studies have suggested that elderly road users need to be the increasing target of road safety policies [6,47,48], especially because, in many countries, the number of such drivers is rising as a result of general population aging. Interestingly, as the number of older drivers becomes more significant, researchers have access to growing amounts of data about this group of drivers, opening up an important line of future research.

Vehicle age is also gaining attention in road safety research, with previous studies suggesting it is positively associated with driver age [12,49,50]. Indeed, vehicles are becoming increasingly safer as a result of technology and safety advances implemented in the new generation of automobiles [51]. Here, we found that the expected proportion of occupants injured by level of severity increases with vehicle age up to 18 years and then remains constant at the highest level. This finding is especially relevant in countries with old fleets of automobiles, such as Spain, where the average age of automobiles has risen from 7.65 in 2002 to 13.49 years in 2021 [52]. In the EU, 2020 data indicate passenger cars are on average 11.5 years old [53].

However, and despite the fact that here we have demonstrated the individual statistical significance of driver age and vehicle age when analyzing the proportion of victims in the vehicle by level of BI severity, we have not observed the individual significance for the joint effect between the two variables (i.e., interaction of driver and vehicle ages). As a result, the hypothesis that longevity in drivers and age in their vehicles are statistically correlated variables is rejected, as is the hypothesis that including the two variables as a covariate has a significant effect on the probability that the injuries resulting from the crash present a certain severity. However, the monitoring of both variables and their effects in forthcoming years constitutes an important line of research, considering the increasing longevity of drivers in countries such as Spain (with a marked expected growth also in the number of people aged 65 and over) and the continuous aging of its vehicle fleet [52].

The rest of our results confirm conclusions previously presented in the literature. Male drivers are associated with accidents involving more serious injuries (for a review, see [4]). Two-wheeled motor vehicles are more likely to be associated with serious or fatal injuries than four-wheeled or heavier vehicles [21,23], which is expected given they offer less protection to riders. Previous studies also suggest that heavy vehicles (pickup trucks, minivans, and sport utility vehicles or SUVs) are safer for their own occupants but cause more damage to the other vehicles involved in a crash [18,24]. A number of studies have found that driving in dark conditions increases expected accident severity [28,29,30]. Sullivan and Flannagan [28] concluded that the risk of fatal injury in pedestrians involved in crashes is 3 to 6.75 times higher in the dark than in daylight. Wanvik [29] found that the risk of injury from accidents in darkness increases on average by 17% on lit rural roadways and by 145% on unlit rural roadways. Uddin and Huynh [30] also confirm the importance of examining lighting conditions on rural and urban roadways as risk factors. Here, we have found that the expected proportions of slight, serious, and fatal injuries following an accident increase when visibility is less than optimum.

We found that non-optimal road surface conditions increase the expected proportion of slightly injured occupants, but in these same conditions the expected proportion of serious and fatal injured victims falls. Although an increase in crash BI severity might be expected with worsening road conditions (bad weather, poor road surfaces, etc.), unobserved factors, such as increased attention to driving, higher traffic density, and higher signaling rates, seem to have an opposite effect. Various studies have shown that the influence of good road conditions on traffic accidents and severity of injuries is unclear, with mixed results having been reported (see, for example, in [54]).

The expected proportion of injured victims is higher on principal and minor roads than on local roads. Although the number of crashes in local areas is usually higher than on arterials and collectors, such accidents are associated with a lower BI severity [55]. The type of crash analyzed has a direct influence on the expected proportion of injured occupants. When a vehicle is involved in a multi-vehicle collision, the expected proportion of injured occupants falls compared to the corresponding proportion for two-vehicle collisions. Collisions involving multiple vehicles (pile-ups) are more frequently rear impact crashes, which are associated with less severe BI outcomes [25,55]. Abu-Zidan and Eid [25] report that injury severity among those involved in front and side impacts was double that of those involved in rear impacts. Likewise, the expected proportion of injured occupants falls when the type of crash is a run-over compared to the corresponding proportion for two-vehicle collisions. Note that in a traffic accident involving a pedestrian, the pedestrian is expected to sustain the highest BI damage [56,57], whereas the occupants of the vehicle (the focus of analysis in this study) are much more protected. Finally, when the vehicle is involved in a rollover, drop, or collision with an object, an increased proportion of injured occupants is expected for all levels of severity. In the literature, when crashes with victims are analyzed, single-vehicle crashes are frequently associated with more severe BI damage than collisions involving two or more vehicles [25,55,58].

The high level of significance of most of our parameter estimates provides a good understanding of the effect of automobile and crash characteristics on the expected number of occupants injured by level of severity. However, our study is not without its limitations. The crash data used in the study are from 2016, which means that the analysis of posterior years would be helpful for understanding the dynamics of elderly drivers and aging vehicles in relation to crash severity. Although we are able to control for the heterogeneity attributable to multiple vehicles being involved in the same crash, other sources of unobserved heterogeneity are not controlled for here. For example, we estimated binomial BI severity models separately for the different levels of BI severity experienced by occupants, but some unobserved factors are likely to impact simultaneously all levels of severity. Additionally, relevant information for explaining the severity of the crash was not always available in the dataset. For instance, the age and position occupied by passengers in the vehicle, the use of safety measures, or the place where the crash occurred have been extensively studied as factors influencing crash severity [33,59,60]. Here, these factors, as well as a lack of information about driving behavior, contribute to unobserved heterogeneity. Indeed, telemetric research points to a close relationship between driving behavior and crash severity [51,60,61,62,63,64]. The incorporation of driving behavior information into the model could differentiate aspects that would further understanding of the influence of traditional risk factors. For example, a better understanding of the driving behavior of old drivers might help to distinguish the proportion of the higher crash severity risk attributable to declining skills and the proportion associated with increased physical frailty.

## 6. Conclusions

Modeling the proportions of non-seriously, seriously, and fatally injured victims in a vehicle involved in a road traffic crash needs to include the dependence between all the vehicles involved in that crash. We have shown that the inclusion of random effects in the regression to capture this phenomenon significantly improves the quality of fit. The driver’s gender, the road type, the type of vehicle involved in the crash, visibility and road conditions, and the type of crash are all factors with explanatory capacity of the expected proportion of occupants injured in each vehicle by level of BI severity. Driver age and vehicle age have a nonlinear influence on severity. We find that the expected proportion of victims increases for both young and old drivers, and vehicle age increases the expected proportion of injured occupants, with the greatest impact being found for cars that are 18 years or older. Yet, we observe no statistical significance of the covariate that reflects interaction between driver age and vehicle age, probably reflecting the great diversity in the age of vehicles driven by older drivers. Accurate modeling of the proportion of injured occupants by level of BI severity that takes into account the dependence between the vehicles involved in the same crash is relevant for traffic authorities in all countries as well as for motor insurance companies who cover the damages of the victims of road traffic crashes. Here, premium design could be improved by including the expected proportion of victims by level of BI severity in the estimation of crash severity.

## Figures and Tables

**Table 1:** Description of dataset of road crashes with complete records.

Description	Number
Total police-reported motor crashes with injured victims	59,040
Total police-reported motor crashes with injured victims involving one vehicle	27,554
Total police-reported motor crashes with injured victims involving two vehicles	27,088
Total police-reported motor crashes with injured victims involving three or more vehicles	4398
Total number of vehicles involved in police-reported motor crashes with injured victims	96,472
Total number of vehicles with injured occupants	55,693
Total number of vehicles without injured occupants (i.e., victims in other vehicles involved)	40,779

**Table 2:** Description of variables.

Name	Categories	Description	Mean *	SD	Min	Max
*Victims (dependent variable)*						
Non-injury		Number of non-injured victims in the vehicle	0.65	0.92	0	58
Slight		Number of slightly injured victims in the vehicle	0.69	0.70	0	45
Serious		Number of seriously injured victims in the vehicle	0.06	0.07	0	21
Fatalities		Number of fatalities in the vehicle	0.01	0.01	0	13
*Vehicle*						
Vehicle age		Age of the vehicle involved in the crash	10.35	5.96	0	30
Vehicle	Car	Cars (category of reference)	0.698	0.459	0	1
	Van	Vans and minibuses	0.070	0.256	0	1
	Motorcycle	Motorcycles	0.120	0.325	0	1
	Moped	Bicycles, Mopeds, and ATVs	0.051	0.219	0	1
	Heavy vehicle	Trucks, tractors, and other heavy vehicles	0.061	0.239	0	1
Occupants		Number of occupants in the vehicle (including the driver)	1.41	1.06	1	61
*Driver*						
Driver age		Age of the driver involved in the crash (divided by 10)	4.14	1.51	1.6	9.50
Gender	Female	Driver is female (category of reference)	0.285	0.451	0	1
	Male	Driver is male	0.715	0.451	0	1
*Crash*						
Illumination	Visibility	Driving with visibility (category of reference)	0.885	0.319	0	1
	No visibility	Driving without appropriate visibility	0.115	0.319	0	1
Road	Local	City streets and township roads (category of reference)	0.477	0.499	0	1
	Principal	Highways, freeways, and other principal arterials	0.172	0.377	0	1
	Minor	Minor arterials and collectors	0.280	0.449	0	1
	Other	Subsidiary roads, unpaved roads, cycling lanes, and others	0.072	0.258	0	1
Condition road	Optimal	Optimal driving conditions of the road surface (category of reference)	0.841	0.366	0	1
	Non-optimal	Non-optimal driving conditions of the road surface (wet, frozen, muddy)	0.159	0.366	0	1
Type of crash	Collision	Collision involving another vehicle (category of reference)	0.619	0.486	0	1
	Pile-up	Multiple vehicle collision	0.078	0.268	0	1
	Run-over	Collision involving a pedestrian or an animal	0.087	0.282	0	1
	Rollover	Rollover, drop, or collision with an object	0.149	0.356	0	1
	Other	Other types of crash	0.067	0.250	0	1

* Relative frequency in % for categorical variables.

**Table 3:** Comparison of binomial regressions without and with random effects.

		Without Random Effects	With Random Effects
Slight victims	AIC	147,940.4	146,992.0
	BIC	148,101.6	147,162.6
Serious victims	AIC	38,800.7	37,443.0
	BIC	38,961.8	37,613.6
Fatalities	AIC	11,752.0	11,430.0
	BIC	11,913.2	11,600.6

**Table 4:** Coefficient estimates of the binomial GLMM according to BI severity of victims.

		Slight		Serious		Fatal	
		Coeff.	SE	Coeff.	SE	Coeff.	SE
*Intercept*		0.1801 ***	0.058	−4.840 ***	0.139	−7.401 ***	0.289
*Gender*	Male	−0.496 ***	0.015	0.213 ***	0.043	0.612 ***	0.097
*Driver age*	Driver age	−0.147 ***	0.023	−0.179 ***	0.053	−0.211 **	0.099
	Squared age	0.008 **	0.002	0.029 ***	0.006	0.048 ***	0.010
*Vehicle age*	Young vehicle	0.021 ***	0.003	0.021 ***	0.007	0.032 **	0.013
	Old vehicle	0.370 ***	0.056	0.482 ***	0.144	0.871 ***	0.264
*Interaction driver and vehicle age*		0.0002	0.001	−0.0001	0.001	−0.002	0.002
*Vehicle*	Van	−0.180 ***	0.025	−0.061	0.077	−0.121	0.140
	Motorcycle	1.616 ***	0.026	2.144 ***	0.043	1.351 ***	0.085
	Moped	1.964 ***	0.042	1.782 ***	0.065	0.874 ***	0.162
	Heavy vehicle	−0.690 ***	0.028	0.080	0.071	0.111	0.123
*Illumination*	No visibility	0.179 ***	0.021	0.502 ***	0.046	0.601 ***	0.078
*Condition road*	Non-optimal	0.262 ***	0.018	−0.358 ***	0.048	−0.338 ***	0.090
*Road*	Principal	0.246 ***	0.020	0.827 ***	0.055	1.356 ***	0.118
	Minor	0.263 ***	0.017	1.191 ***	0.043	1.721 ***	0.100
	Other	0.127 ***	0.027	0.503 ***	0.071	0.967 ***	0.151
*Crash*	Pile-up	−0.090 ***	0.025	−0.982 ***	0.108	−1.279 ***	0.238
	Run-over	−2.342 ***	0.039	−1.117 ***	0.126	−0.898 ***	0.260
	Rollover	0.700 ***	0.020	0.654 ***	0.040	0.809 ***	0.074
	Other	0.455 ***	0.026	0.581 ***	0.056	0.745 ***	0.099
SD (Random effect)		0.526		1.453		1.551	
AIC		146,931.3		37,419.4		11,404.6	
BIC		147,130.3		37,618.5		11,603.7	

Note: *** *p*-value < 0.001; ** *p*-value < 0.05; * *p*-value < 0.10.

## Data Availability

Data were provided by Spanish Transport Authority (Dirección General de Tráfico) upon request.

## Appendix A

**Table A1:** Comparison of binomial regressions with and without random effects.

		Slight Victims				Serious Victims				Fatal Victims			
		Generalized Linear Model		Generalized Linear Mixed Model		Generalized Linear Model		Generalized Linear Mixed Model		Generalized Linear Model		Generalized Linear Mixed Model	
		Coeff.	Std. Error	Coeff.	Std. Error	Coeff.	Std. Error	Coeff.	Std. Error	Coeff.	Std. Error	Coeff.	Std. Error
*Intercept*		0.071 **	0.023	0.051 **	0.024	−5.085 ***	0.061	−5.38 ***	0.071	−8.24 ***	0.148	−8.352 ***	0.158
*Gender*	Male	−0.443 ***	0.013	−0.467 ***	0.014	0.294 ***	0.038	0.235 ***	0.043	0.7 ***	0.092	0.657 ***	0.097
*Driver age*		−0.007 ***	0.001	−0.007 ***	0.000	0.008 ***	0.001	0.008 ***	0.001	0.023 ***	0.002	0.023 ***	0.002
*Vehicle age*		0.019 ***	0.001	0.018 ***	0.001	0.017 ***	0.002	0.022 ***	0.003	0.026 ***	0.005	0.03 ***	0.005
*Vehicle*	Van	−0.160 ***	0.023	−0.178 ***	0.025	−0.092	0.067	−0.085	0.076	−0.157	0.128	−0.17	0.139
	Motorcycle	1.406 ***	0.023	1.519 ***	0.024	1.87 ***	0.036	2.103 ***	0.042	1.163 ***	0.076	1.258 ***	0.083
	Moped	1.788 ***	0.039	1.913 ***	0.04	1.613 ***	0.055	1.812 ***	0.064	0.809 ***	0.151	0.893 ***	0.16
	Heavy vehicles	−0.671 ***	0.025	−0.668 ***	0.027	0.109*	0.059	0.031	0.071	0.167	0.105	0.014	0.121
*Illumination*	No visibility	0.147 ***	0.018	0.169 ***	0.02	0.48 ***	0.037	0.498 ***	0.046	0.611 ***	0.068	0.594 ***	0.078
*Condition road*	Non-optimal	0.216 ***	0.016	0.248 ***	0.018	−0.389 ***	0.041	−0.362 ***	0.048	−0.377 ***	0.082	−0.342 ***	0.09
*Road*	Principal	0.152 ***	0.017	0.230 ***	0.019	0.783 ***	0.046	0.816 ***	0.055	1.439 ***	0.11	1.337 ***	0.118
	Minor	0.207 ***	0.015	0.250 ***	0.016	1.18 ***	0.036	1.194 ***	0.043	1.799 ***	0.096	1.729 ***	0.1
	Others	0.094 ***	0.024	0.121 ***	0.026	0.473 ***	0.06	0.505 ***	0.071	1.013 ***	0.143	0.974 ***	0.15
*Crash*	Pile-up	−0.103 ***	0.021	−0.088 ***	0.025	−0.971 ***	0.091	−0.992 ***	0.108	−1.345 ***	0.22	−1.302 ***	0.237
	Run over	−2.201 ***	0.037	−2.221 ***	0.039	−1.207 ***	0.118	−1.112 ***	0.127	−1.011 ***	0.255	−0.893 ***	0.26
	Rollover	0.669 ***	0.018	0.666 ***	0.019	0.598 ***	0.033	0.66 ***	0.04	0.77 ***	0.067	0.823 ***	0.074
	Others	0.419 ***	0.023	0.434 ***	0.025	0.543 ***	0.046	0.591 ***	0.056	0.768 ***	0.087	0.764 ***	0.099
Standard deviation (Random effect)					0.513				1.456				1.546
AIC			147,940.4		146,992.0		38,800.7		37,443.0		11,752.0		11,430.0
BIC			148,101.6		147,162.6		38,961.8		37,613.6		11,913.2		11,600.6

*** *p*-value<0.001; ** *p*-value<0.05; * *p*-value<0.10.

## Appendix B. Semiparametric Binomial Regression Model

**Table A2:** Coefficient estimates according to BI severity of victims.

		Slight Victims		Serious Victims		Fatal Victims	
		Coeff.	Std. Error	Coeff.	Std. Error	Coeff.	Std. Error
*Intercept*		−0.065 ***	0.014	−2.418 ***	0.020	−3.235 ***	0.042
*Gender*	Male	−0.494 ***	0.014	0.114 ***	0.017	0.243 ***	0.034
*Vehicle*	Van	−0.182 ***	0.025	−0.029	0.030	−0.043	0.050
	Motorcycle	1.620 ***	0.025	0.968 ***	0.019	0.529 ***	0.032
	Moped	2.036 ***	0.042	0.776 ***	0.028	0.310 ***	0.061
	Heavy vehicles	−0.685 ***	0.027	0.075 **	0.027	0.104 *	0.042
*Illumination*	No visibility	0.178 ***	0.020	0.228 ***	0.018	0.247 ***	0.030
*Condition road*	Non-optimal	0.262 ***	0.018	−0.170 ***	0.019	−0.146 ***	0.032
*Road*	Principal	0.244 ***	0.019	0.377 ***	0.021	0.534 ***	0.040
	Minor	0.262 ***	0.016	0.582 ***	0.017	0.686 ***	0.034
	Others	0.125 ***	0.026	0.234 ***	0.028	0.362 ***	0.052
*Crash*	Pile-up	−0.090 ***	0.024	−0.374 ***	0.035	−0.437 ***	0.071
	Run over	−2.330 ***	0.040	−0.504 ***	0.048	−0.336 ***	0.086
	Rollover	0.699 ***	0.019	0.66 ***	0.04	0.315 ***	0.027
	Others	0.451 ***	0.025	0.591 ***	0.056	0.303 ***	0.004
Standard deviation (Random effects)			0.5250		-^ (a)^		-^ (a)^
Approximate significance of smooth spline terms							
		edf ^(b)^	Chi.sq ^(c)^	edf ^(b)^	Chi.sq ^(c)^	edf ^(b)^	Chi.sq ^(c)^
	*Driver age*	6.089	351.5 ***	4.797	123.97 ***	3.842	213.22 ***
	Vehicle age	4.379	325.6 ***	5.085	56.57 ***	4.030	31.53 ***
AIC		146882.35		38560.37		11697.33	

*** *p*-value<0.001; ** *p*-value<0.05; * *p*-value<0.10. ^(a)^ The estimated variance matrix was not semidefinite positive in the semiparametric binomial GLMM model. Reported results are from the semiparametric binomial GLM model. ^(b)^ edf: The effective degrees of freedom (edf) estimated from generalized additive models are a proxy for the degree of non-linearity in covariate-response relationships. An edf of 1 is equivalent to a linear relationship. An edf > 1 and ≤ 2 is a weakly non-linear relationship, and an edf > 2 indicates a highly non-linear relationship (Zuur et al. 2009). ^(c)^ Chi.sq: Chi-squared test for the removal of the smooth covariate in the fitting.
